# Medical Students Show Lower Physical Activity Levels and Higher Anxiety Than Physical Education Students: A Cross-Sectional Study During the COVID-19 Pandemic

**DOI:** 10.3389/fpsyt.2021.804967

**Published:** 2021-12-16

**Authors:** Karla Cardoso de Souza, Tassia Barcelos Mendes, Tabatah Hellen Santos Gomes, Ariana Aline da Silva, Luiz Henrique da Silva Nali, Andre Luis Lacerda Bachi, Fabricio Eduardo Rossi, Saulo Gil, Carolina Nunes França, Lucas Melo Neves

**Affiliations:** ^1^Santo Amaro University, São Paulo, Brazil; ^2^Post-Graduation Program in Health Sciences, Santo Amaro University, São Paulo, Brazil; ^3^Immunometabolism of Skeletal Muscle and Exercise Research Group, Department of Physical Education and Professor at Graduate Program in Science and Health, Federal University of Piauí (UFPI), Teresina-PI, Brazil; ^4^Bipolar Disorder Program (PROMAN), Department of Psychiatry, University of São Paulo Medical School, São Paulo, Brazil

**Keywords:** sedentary behavior, university, exercise, coronavirus – COVID-19, student

## Abstract

**Objective:** This study aimed to compare the time in physical activity (PA) [light (LPA), moderate and vigorous (MVPA)] and sedentary behavior (SB) (weekdays, weekends, or both) between Medical (MED) and Physical Education (PE) students who underwent remote classes imposed by the COVID-19. In addition, we compared symptoms of depression and anxiety and sleep quality.

**Methods:** A cross-sectional study (272 MED and 95 PE students). The International Physical Activity Questionnaire (IPAQ), Beck Inventory (Anxiety, Depression), and Pittsburgh Sleep Quality were used to assess PA and SB, anxiety and depression symptoms, and quality of sleep, respectively. The data are presented as median and interquartile intervals 25–75.

**Results:** We observed statistically significant differences between MED and PE students for MVPA [MED: 165 min per week (0–360) vs. PE: 420 min per week (180–670), *p* < 0.001], SB Total [MED: 10 h per day (8–12) vs. PE: 7 h per day (5–10), *p* < 0.001)], and anxiety symptoms [MED: 13 points (5–23) vs. PE: six points (2–16), *p* < 0.001)].

**Conclusion:** Together, our findings indicate that MED students spent less time in MVPA and more time in SB than PE students. MED students also presented worse mental health in the pandemic situation imposed by the COVID-19.

## Introduction

The COVID-19 pandemic (1) led the world population to adopt preventive measures, such as hygiene habits, and to maintain social distancing and home isolation to control the spread of the virus. In this scenario, common activities were drastically affected, such as work routine, (2) leisure-related activities (3), and regular classes at schools and universities (4).

Home isolation may impact levels of physical activity (PA, defined as activity that gets your body moving, to increase energy expenditure above resting levels) (5) and sedentary behavior (SB, defined as waking behavior characterized by an energy expenditure ≤ of 1.5 metabolic equivalents, while in a sitting, reclining, or lying posture) (6). Indeed, recent data showed that PA and SB were drastically affected by the COVID-19 pandemic (7). This scenario was inevitable due to the lack of vaccinations early in the pandemic.

PA and SB are associated with mental health and sleep quality (8). For example, high levels of PA are related to lower symptoms of depression and anxiety (9) and greater sleep quality (10). On the other hand, elevated SB is associated with depression and anxiety symptoms (11). Furthermore, home isolation due to the COVID-19 pandemic has been related to worsening mental health in the general population (12, 13). Moreover, negative mental alterations such as depression and anxiety symptoms may lead to a poor physical activity-related lifestyle (e.g., lower PA and higher SB), establishing a “vicious cycle” among these conditions (11, 14).

Students were drastically affected by the COVID-19 pandemic since education systems adopted remote classes. Under normal conditions, most university students already did not meet the recommendation of PA and spent approximately 7–8 h per day in SB (15). In the same way, we observed that more than two thirds of Brazilian medical students did not meet the recommendation of PA and spent more than 8 h per day in SB during the pandemic (14). This poor physical activity-related lifestyle may impair sleep quality and increase the depression and anxiety symptoms commonly observed in MED students (16, 17). Interestingly, most Brazilian physical education (PE) students meet physical activity recommendations (18). In fact, a recent meta-analysis (15 studies, 3,245 students) indicates that between two-thirds and all (71 to 100%) Brazilian PE meet PA recommendations.

Although it is still possible to speculate that the PA and SB of PE students were negatively influenced during the COVID-19 pandemic, in the same way as MED students, due to the change from regular classes to remote classes. On the other hand, despite the possible negative impact of the pandemic on PA and SB, engagement of PE students in the practical demands related to the physical education classes characteristic of their course and associated internships, may confer some advantage compared to MED students. However, this assumption still needs to be elucidated.

Another important point related to the routines of MED students is activities to assist the population during a pandemic, particularly students enrolled in the second half of the course (6^th^ to 12^th^ semesters). Furthermore, lack of resources and staffing, coupled with reduced staff numbers through contamination with the virus, may have further increased the stressful situations related to medical work demand (19). In this scenario, it is reasonable to assume that MED students from different semesters may present distinct mental health, as well as different times of PA and SB due to time taken with patient care. Therefore, it is feasible to compare the first half of the course with the second half of the course.

Thus, the current study aimed to compare the levels of PA [light (LPA), moderate and vigorous (MVPA)] and SB (on weekdays, weekends, or both) between MED and PE students who underwent remote classes, imposed due to the COVID-19 pandemic. In addition, we compared depression and anxiety symptoms and sleep quality of MED and PE students, and the first and second halves of the courses. We hypothesized that MED students would spend less time in PA and more time in SB and demonstrate worse mental health than PE students, with differences between the first and second halves of the MED course.

## Methods

### Study Design and Participants

This is a cross-sectional study, which was approved by the local Ethics and Research Committee (approval number: 4.049.214), and followed the precepts of the Declaration of Helsinki. The data were collected between September 2020 and February 2021. All students agreed and signed the consent form. Students aged ≥18 years from MED (1^st^ to 12^th^ semester) and PE (1^st^ to the 8^th^ semester) were invited to participate in the study. Participants were contacted via message application or e-mail. If the participant agreed to participate, they were sent an online form containing the consent form and questionnaires to assess demographic characteristics, PA, SB, depression and anxiety symptoms, and sleep quality. For better comprehension of the collected information, initially, the student representative of the groups (WhatsApp group administrator) sent the research questionnaire link directly to the WhatsApp group. After 4 days, the authors (TB and KC) were added to these WhatsApp groups and again sent the research questionnaire link and were available to clarify any doubts. Of 1,400 students from 30 distinct classes contacted, 367 students (MED: 272 = 27% of all MED students; PE: 95 = 24% of all PE students) answered the questionnaire. No students directly stated that they did not want to participate in the survey. Despite the groups being unbalanced, we emphasize that, in percentage terms, ~25% of the students in each course were evaluated.

### Demographic Characteristics and Self-Perception During the COVID-19 Pandemic

A questionnaire was included to collect data on age, sex, semester of the course, the city lived in before enrolling on the course, the practice of a physical exercise program, COVID-19 diagnosis, use of tobacco and alcohol, and questions about self-perception of worsening of the level of PA and SB during the period of the COVID-19 pandemic.

### Sample Size Calculation

The sample size was determined using G-Power software (version 3.1.2 – Universitat Kiel, Germany), inputting an α error (0.05) and power (1 – β error = 0.90). Since the literature is scarce concerning comparisons of PA and SB between MED and PE students, we assumed an arbitrary moderate effect size (Cohen' d) of 0.4. Calculations were based on an independent *t*-test, and the total sample size was determined 180 patients (i.e., 90 students in each group).

### Measures

#### Physical Activity and Sedentary Behavior Assessment

The times of PA (LPA, MVPA) and SB (weekdays, weekends, or both) were assessed by the International Physical Activity Questionnaire (IPAQ), which contains eight questions about PA and SB considering the routine of the previous seven days. This tool is widely used and validated for the Brazilian population (20). The IPAQ shows good reliability (Spearman correlation coefficients = 0.80) and test–retest reliability (Spearman correlation coefficients = 0.80), and presents high correlations with other measures of physical activity (accelerometer = 0.70 to 0.80) (21).

#### Anxiety and Depression Symptom Assessment

Anxiety and depression symptoms were assessed by the Beck Anxiety Inventory (BAI) and Beck Depressive Inventory (BDI) (22, 23), which are composed of 21 multiple-choice statements, each with four possible responses (0–3). Thus, the final score ranges from 0 to 63 points. The BAI shows good reliability (Cronbach's alpha coefficient = 0.95) and test–retest reliability (Pearson's r = 0.73 to 0.96), and presents high correlations with other measures of anxiety (State-Trait Anxiety Inventory = 0.58; Diary Anxiety = 0.54) (24). The BAI is a widely used tool, validated for the Brazilian population (25). The cut-off point adopted to identify symptoms of low and high anxiety followed that shown in a previous study (26): <13 points (low anxiety symptoms) and ≥ 13 points (high anxiety symptoms).

The BDI is also a validated tool for the Brazilian population (27) and shows good reliability (Cronbach's alpha coefficient = 0.85) and test–retest reliability (Pearson's r = 0.76), and presents high correlations with other measures of depression, including the Center for Epidemiologic Studies of Depression (Pearson's r = 0.66–0.86) and Hamilton Rating Scale for Depression (Pearson's r = 0.66–0.75) (28). The cut-off point adopted to identify symptoms of low and high depression followed that shown in previous studies (29): <10 points (symptoms of low depression) and ≥ 10 points (symptoms of high depression).

#### Sleep Assessment

Sleep quality was assessed by the Pittsburgh Sleep Quality Index (PSQI), validated for the Brazilian population (30). The PSQI contains questions about the subject's sleep habits during the previous month. For example, bedtime, time to fall asleep, time to wake up, and actual h of sleep, in addition to the frequency (1, 2, or 3 times a week) of difficulty falling asleep in 30 min, waking up at night/dawn, getting up at night to go to the bathroom, difficulty breathing, coughing, snoring, cold, heat, nightmares, other reasons. The final score ranges from 0 to 21 points. The measure consists of 19 individual items, creating seven components (Subjective sleep quality; Sleep latency; Sleep duration; Habitual sleep efficiency; Sleep disturbances; Use of sleeping medication; Daytime dysfunction) and a Global PSQI score (sum of the 7 components). The PSQI shows good reliability (Cronbach's alpha coefficient = 0.82) (31) and test–retest reliability (a intraclass correlation coefficient = 0.81) (32), and presents high correlations with other measures of sleep quality (i.e., clinical diagnosis of insomnia, the ISI score, some variables of polysomnography) (32). In addition, the most commonly reported cut-off point for MS in a recent meta-analysis (33) was adopted: <6 points (good sleep quality) and ≥6 points (poor sleep quality).

### Statistical Analysis

Data are presented as mean (standard deviation), or median and interquartile range (IQR) for continuous variables. Categorical variables are shown as absolute and frequencies (%). Normality and equality of variances were tested using the Kolmogorov-Smirnov test and Levene test, respectively. Non-parametric data are shown as median and interquartile range (IQR: 25 – 75). The Mann-Whitney test was used to assess differences between groups (PE and MED) and between the first and second halves of the courses (1^st^ to 4^th^ and 5^th^ to 8^th^ in PE; 1^st^ to 6^th^ and 7^th^ to 12^th^ in MED). Logistic regression models were used to evaluate the association between course (MED or PE), and dichotomous variables: MVPA (<300 min per week vs ≥300 min per week), SB (< than 8 h vs ≥ than 8 h). Additionally, the association between course (MED or PE) and continues variables: anxiety symptoms (points of BAI), depression symptoms (points of BDI), and sleep quality (points of PSQI) were evaluated. Results from the logistic regression models were presented as odds ratios (ORs). The presence of relationships between numerical variables (MVPA, SB, Anxiety and Depression symptoms, and, Sleep Quality), was examined with the Spearman Correlation Test. Data were analyzed using IBM SPSS Statistics, version 22 (SPSS Inc., Chicago, IL, USA). The significance level was set at P ≤ 0.05.

## Results

Table 1 details the main sample characteristics. In brief, 1,400 students from 30 distinct classes were contacted, of which 367 students (MED: 272; PE: 95) answered the questionnaire and were included in the analysis. The sample of MED students was predominantly composed of females (79%), while the sample of PE students was composed mainly of males (63%). The median ages for MED and PE students were 21 and 25 years, respectively. Sixty-seven percent (67%) of the MED students were inactive (<300 min per week), compared to only thirty-four percent (34%) of the PE students. Seventy-nine percent (79%) of the MED students and 57% of the PE students reported spending more than 8 h in SB.

**Table 1:** Sample characteristics of medical (MED) and physical education (PE) students.

**Variable**	**MED (*N* = 272)**	**PE (*n* = 95)**
Age (years)	21.0 (19.3–23.0)	25.3 (20.0–30.0)
Sex
Female	215 (79%)	35 (36.8%)
Male	57 (21%)	60 (63.2%)
The first half of the courses
(1^st^ to 4^th^ PE or 1^st^ to 6^th^ MED)	221 (81.2%)	53 (55.8%)
The second half of the courses
(5^th^ to 8^th^ PE or 7^th^ to 12^th^ MED)	51 (18.8%)	42 (44.2%)
MVPA
Physically inactive (<300 min per week)	184 (67.7%)	33 (34.8%)
Physically active (≥300 min per week)	89 (32.3%)	62 (65.2%)
SB ≥ than 8 h
Yes	216 (79.4%)	55 (57.9%)
No	66 (20.6%)	40 (42.1%)
Have you performed systematic physical exercises in the last 6 months? (This includes weight training, swimming, dancing, or any other type of activities, performed as part of your routine)		
Yes	181 (66.5%)	74 (77.9%)
No	91 (33.5%)	21 (22.1%)
Anxiety symptoms
0 to 12 points (low anxiety symptoms ^*^)	135 (49.6%)	73 (76.8%)
>12 points (high anxiety symptoms ^*^)	137 (50.4%)	22 (23.2%)
Depression symptoms
0 to 10 points (low depression symptoms ^**^)	132 (48.5%)	59 (62.1%)
>10 points (elevated depression symptoms ^**^)	140 (51.5%)	36 (37.9%)
Sleep quality
Good sleep quality (<6 points) ^***^	125 (46.0%)	53 (55.8%)
Poor sleep quality (≥6 points) ^***^	147 (54.0%)	42 (44.2%)
Have you had a COVID-19 diagnosis?
Yes	30 (11.0%)	16 (16.8%)
No	242 (89.0%)	79 (83.2%)
Do you consider that the COVID-19 pandemic changed your level of physical activity and SB?		
Yes	226 (83.1%)	64 (67.4%)
No	46 (16.9%)	31 (32.6%)
Do you consider that the COVID-19 pandemic changed your level of anxiety symptoms?		
Yes	220 (80.9%)	56 (58.9%)
No	52 (19.1%)	39 (41.1%)
Do you consider that the COVID-19 pandemic changed your level of depression symptoms?		
Yes	150 (55.1%)	32 (33.7%)
No	122 (44.9%)	63 (66.3%)
Do you use cigarettes?
Yes	40 (14.7%)	6 (6.3%)
No	232 (85.3%)	89 (93.7%)
Do you use alcohol?
Yes	180 (66.2%)	41 (43.2%)
No	92 (33.8%)	54 (56.8%)

*SB, sedentary behavior; MVPA, moderate and vigorous physical activity;*

* Based on the cut-off of Sæmundsson et al. (26)

** *Based on the cut-off of Gomes et al. (27)*

*** *Based on the cut-off of Rao et al. (33)*

Table 2 presents a comparison between MED and PE students regarding the sleep variable PSQI. Considering the Global PSQI score, we did not verify a significant difference between the groups (*p* > 0.05). However, when comparing specific components of PSQI, we observed greater scores in sleep duration and habitual sleep efficiency for MED students compared to PE students (both *p* < 0.05). On the other hand, PE students showed greater scores for use of sleeping medication and daytime dysfunction than MED students (both *p* < 0.05).

**Table 2:** Comparison between Medicine and Physical Education students regarding the sleep variable Pittsburgh Sleep Quality Index.

**PSQI Components**	**Medicine students (*N* = 272) Median – Q1-Q3**	**Physical Education students (*N* = 95) Median – Q1-Q3**	***p*-value**
Subjective sleep quality	1 (1–2)	1 (1–2)	0.707
Sleep latency	2 (1–2)	1 (1–2)	0.173
Sleep duration	1 (0–2)	2 (0–2)	0.058
Habitual sleep efficiency	0 (0–1)	0 (0–1)	0.022
Sleep disturbances	1 (1–1)	1 (1–1)	0.569
Use of sleeping medication	0 (0–0)	0 (0–0)	<0.001
Daytime dysfunction	1 (1–2)	1 (0–2)	<0.002
Global PSQI score	7 (4–10)	7 (4–10)	0.509

*All PSQI components are presented in total points (0–3). Global PSQI score presented in total points (0–21). PSQI, Pittsburgh Sleep Quality Index*.

**Figure 1:**
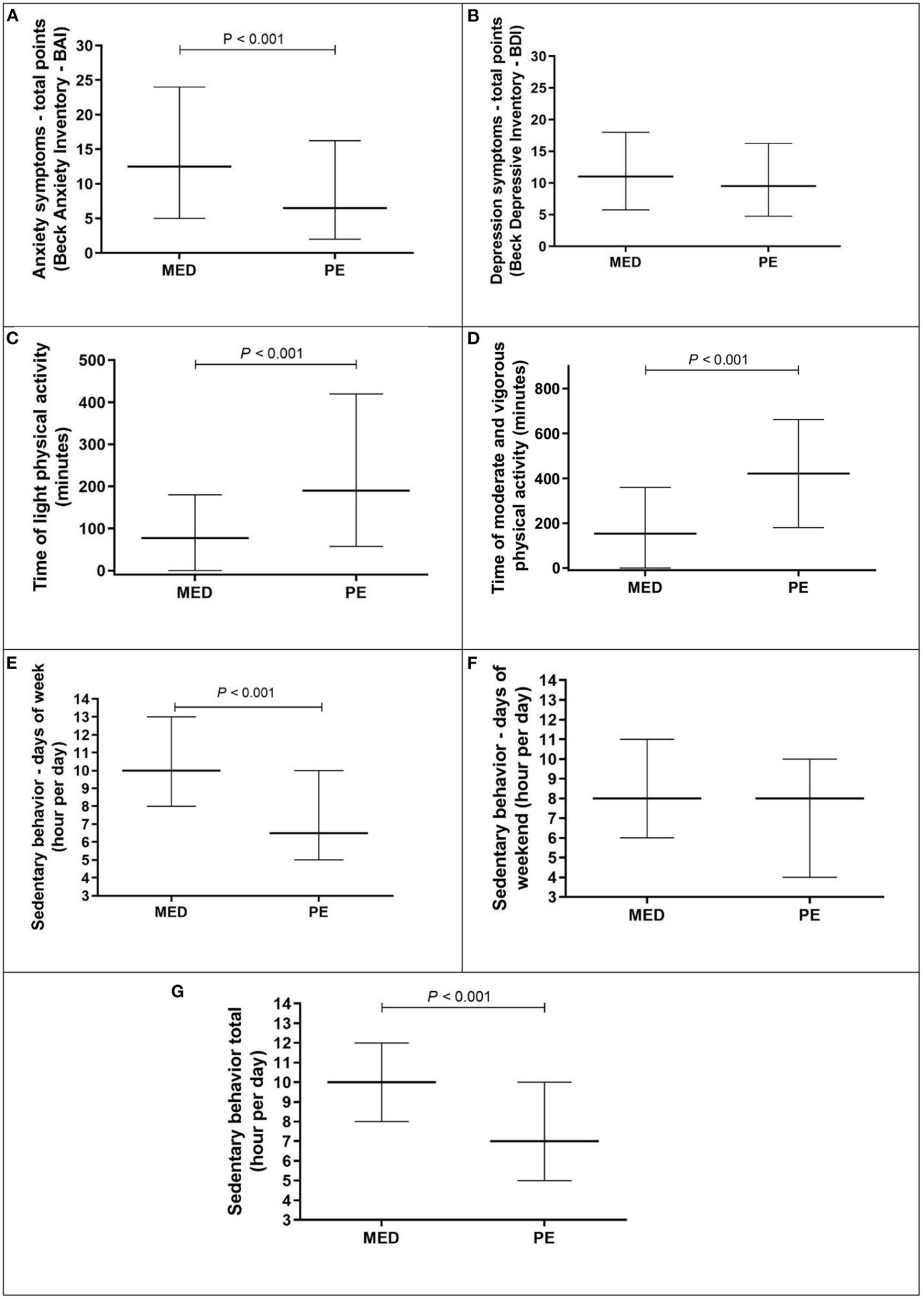
Comparison between medical (MED) and physical education (PE) student regarding anxiety symptoms, depression symptoms, time of light, moderate and vigorous physical activity, and time of sedentary behavior (week, weekend, and total). Data present in median and interval interquartile (25–75). Anxiety Symptoms and Depression symptoms present in total points (0–63). LPA and MVPA are present in minutes per week. Sedentary behavior of week, weekend, and Total are present in hour per day. **Panel A** - Anxiety symptoms (BAI); **panel B** - Depression symptoms (BDI); **panel C** - Light physical activity; **panel D** - Moderate and vigorous physical activity; **panel E** - Sedentary behavior days of week; **panel F** - Sedentary behavior days of the weekend; **panel G** - Sedentary behavior total.

Figure 1 presents the comparisons between MED and PE students regarding: panel A - Anxiety symptoms (BAI); panel B - Depression symptoms (BDI); panel C - Light physical activity; panel D - Moderate and vigorous physical activity; panel E - Sedentary behavior days of week; panel F - Sedentary behavior days of the weekend; panel G - Sedentary behavior total.

The MED students presented worse scores related to anxiety symptoms [median above the cut-off for high anxiety symptoms – >12 points - SÆMUNDSSON et al. (2011)] (26), physical activity - LPA and MVPA (median lower than recommendation of 300 min per week), and SB days of week and total (more than 8 h per day) in comparison to PE students.

Table 3 presents a comparison (Mann-Whitney Test) between the first and second halves of the courses (1^st^ to 4^th^ and 5^th^ to 8^th^ in PE; 1^st^ to 6^th^ and 7^th^ to 12^th^ in MED) regarding anxiety symptoms (BAI), depression symptoms (BDI), and variables related to the level of physical activity (LPA and MVPA) and SB (week, weekend, and total).

**Table 3:** Comparisons (Mann-Whitney Test) between the first and second halves of the courses (1st to 4th and 5th to 8th in PE; 1st to 6th and 7th to 12th in MED) regarding anxiety symptoms (Beck Anxiety Inventory - BAI), depression symptoms (Beck Depressive Inventory - BDI), level of physical activity and sedentary behavior (IPAQ).

	**Medicine students** **(*****N*** **=** **272)** **Median – Q1-Q3**		**p-value**	**Physical Education students** **(*****N*** **=** **95)** **Median – Q1-Q3**		***p*-value**
	**The first half of the course (*N* = 221)**	**The second half of the course (*N* = 51)**		**The first half of the course (*N* = 53)**	**The second half of the course (*N* = 42)**	
Anxiety Symptoms	13 (6–24)	11 (4–21)	0.079	10 (2–19)	5(2–12)	0.074
Depression symptoms	11 (5–18)	12 (6–16)	0.746	11 (4–18)	9 (4–13)	0.329
LPA	90 (50–240)	60 (40–150)	0.451	150 (80–410)	180 (82–420)	0.735
MVPA	160 (80–360)	180 (450)	0.652	420 (180–725)	470 (202–660)	0.831
SB week	10 (8–13)	10 (8–12)	0.723	6 (4–10)	7 (5–10)	0.299
SB weekend	8 (5–11)	10 (7–12)	0.057	6 (4–10)	8 (4–13)	0.442
SB Total	9 (7–12)	10 (8–13)	0.653	6 (4–9)	7 (5–10)	0.221
Subjective sleep quality	1 (1–2)	1 (1–2)	0.363	1 (1–2)	1 (1–2)	0.063
Sleep latency	2 (1–2)	1 (1–3)	0.846	2 (1–2)	1 (0–2)	0.033
Sleep duration	1 (1–2)	1 (1–2)	0.152	2 (1–2)	2 (1–2)	0.633
Habitual sleep efficiency	1 (1–2)	1 (1–2)	0.488	1 (0–1)	1 (0–1)	0.658
Sleep disturbances	1 (1–1)	1 (1–1)	0.389	1 (1–2)	1 (1–1)	0.003
Use of sleeping medication	1 (1–2)	1 (1–2)	0.703	1 (0–2)	1(0–2)	0.927
Daytime dysfunction	1 (1–2)	1 (1–2)	0.746	1 (0–2)	1(0–1)	0.314
Global PSQI score	7 (4–10)	7 (4–11)	0.079	7 (5–10)	6 (3–9)	0.069

*Anxiety Symptoms and Depression symptoms presented in total points (0–63). LPA and MVPA presented in minutes per week. SB week, SB weekend, and SB Total, presented in an hours per day. All PSQI components are presented in total points (0–3). Global PSQI score presented in total points (0–21). LPA, light physical activity; MVPA, moderate and vigorous physical activity; SB, sedentary behavior; PSQI, Pittsburgh Sleep Quality Index*.

The first and second halves of the courses (1^st^ to 4^th^ and 5^th^ to 8^th^ in PE; 1^st^ to 6^th^ and 7^th^ to 12^th^ in MED) did not present differences for anxiety and depression symptoms, physical activity (LPA and MVPA), and SB (week, weekend, and total). Furthermore, for sleep quality (PSQI), we did not verify differences between the first and second halves of the course in MED (1^st^ to 6^th^ and 7^th^ to 12^th^), but differences were verified between the first and second halves of the course in PE (1^st^ to 4^th^ and 5^th^ to 8^th^), for sleep latency and sleep disturbances.

Table 4 presents a logistic regression association of course (MED or PE) and meeting the MVPA recommendation, accumulated <8 h of SB, High Anxiety symptoms (<12 points), High Depression symptoms (<10 points), and Poor Sleep Quality (≥6 points).

**Table 4:** Regression logistic association of course (MED or PE) and meet MVPA recommendation, accumulated <8 h of SB, Anxiety symptoms (<12 points), Depression symptoms (<10 points), and Poor Sleep Quality (≥6 points).

	**OR**	**95% CI**	***p***	
MVPA	3.87	2.27	6.59	<0.001
Sedentary behavior	0.28	0.17	0.47	<0.001
Anxiety symptoms	0.58	0.31	1.07	0.08
Depression symptoms	1.27	0.67	2.39	0.46
Sleep Quality	1.27	0.70	2.31	0.43

*CI, confidence interval; OR, odds ratio. Odds of those who referred MVPA ≥ 300 min per week vs. those that referred MVPA <300 min per week having depressive symptoms (BDI>12 points) and anxiety symptoms (BAI>10 points), Sleep quality (PSQI ≥ 6 points*.

The course was associated with an increase in meeting MVPA recommendations (OR = 3.87, 95%CI 2.27–6.59 *p* < 0.001) and a decrease in reporting >8 h per day of SB (OR = 0.28, 95%CI 0.17–0.47 *p* < 0.001.

Table 5 presents a relationship (Spearman Correlation) between MVPA, SB, Anxiety symptoms, Depression symptoms, and Sleep Quality.

**Table 5:** Relationship (Spearman Correlation) between MVPA, SB, Anxiety symptoms, Depression symptoms, and Sleep Quality.

	**MVPA**	**Sedentary behavior**	**Anxiety symptoms**	**Depression symptoms**	**Sleep Quality**
MVPA	—	−0.20^**^	−0.22^**^	−0.23^**^	−0.10
Sedentary behavior	−0.20^**^	—	0.17^**^	0.13^*^	0.01
Anxiety symptoms	−0.22^**^	0.17^**^	—	0.67^**^	0.55^**^
Depression symptoms	−0.23^**^	0.13^**^	0.69^**^	—	0.58^**^
Sleep Quality	**–**0.10	0.01	0.55^**^	0.58^**^	—

*Spearman correlation*.

* *indicates that the correlation is significant; p < 0.05*.

** *indicates that the correlation is significant; p < 0.001. MVPA, Moderate and vigorous physical activity*.

Significant correlations were observed of MVPA and Depression symptoms (−0.23 *p* < 0.001), SB and Anxiety symptoms (0.17 *p* < 0.001), Anxiety symptoms and Depression symptoms (0.67 *p* < 0.001), Depression symptoms and Sleep quality (0.58 *p* < 0.001), and Sleep Quality and Anxiety symptoms (0.55 *p* < 0.001).

## Discussion

The current study aimed to compare MED and PE students during the COVID-19 pandemic, considering the time of PA (LPA and MVPA) and SB (week, weekend, and both), symptoms of anxiety and depression, and sleep quality. Our main finding indicates that MED students perform shorter MVPA, longer SB, and have more anxiety symptoms than PE students. In addition, we observed better scores of sleep duration and habitual sleep efficiency and worse scores of use of sleeping medication and daytime dysfunction in MED students than PE students. However, the Global PSQI score was similar between groups. We also highlight the lack of differences between the first and second halves of the course in MED (1^st^ to 6^th^ semesters and. 7^th^ to 12^th^ semesters) and in PE (1^st^ to 4^th^ semesters and 5^th^ to 8^th^ semesters) considering the symptoms of anxiety and depression, LPA, MVPA, and SB (week, weekend, and total). In addition, no differences were observed for initial and final semesters in sleep quality (PSQI) for MED students, while for PE students, initial and final semesters presented differences in sleep latency and sleep disturbances components, but not for the Global PSQI score. PE students present ORs (OR = 3.87, 95%CI 2.27–6.59 *p* < 0.001) of meeting MVPA recommendations. Significant correlations were verified of MVPA and Depression symptoms and Anxiety symptoms, as well as Anxiety symptoms and Depression symptoms, Depression symptoms and Sleep quality, and Sleep Quality and Anxiety symptoms.

The respective prevalences of elevated anxiety and depression symptoms in MED (50.4 and 51.5%) were higher than the prevalence of anxiety (31.9%) summarized in 17 studies (*n* = 63,439) and depression (33.7%) summarized in 14 studies (*n* = 44,531) (34). The absolute value prevalence of elevated anxiety and depression symptoms in PE (23.2 and 37.9%) was similar or better than presented in the meta-analysis, which could have been influenced by the MVPA and SB levels.

The relationship between lower MVPA and higher symptoms of depression and anxiety in the general population is well-described in the literature (35, 36) and in MED students (37), suggesting that inactive MED students may present higher anxiety and/or depressive symptoms. Our findings confirm, at least partially, our initial hypothesis as we observed elevated physical inactivity (lower MVPA and higher SB) and higher anxiety symptoms in MED students than PE students, who were more active and reported fewer symptoms of anxiety. Some studies are available that evaluate the duration of PA and SB, and a meta-analysis including 15 studies carried out with PE students indicated that they meet the MVPA recommendation. However, this situation is not repeated with MED students, since more than two thirds of our group did not meet the MVPA recommendations (14). Although it was not our objective to investigate the reasons for these differences, it should be emphasized that PE students, in addition to having a lower workload than MED students (approximately half the workload), have curricular components with a strong practical character, which preserve an active lifestyle (38). In contrast, medical students spend more time in SB and perform less physical activity (39), which is justified in part due to a more significant load of theoretical classes.

Additionally, we highlight the extensive literature indicating that MED students and even medical professionals present impairments in mental health (14, 40). Although this situation may be partially attributed to the high SB time and reduced MVPA typical of MED students, associated with high study and workloads, and pressure to succeed from society and family, it may contribute to impairments in mental health.

Especially during the pandemic, a systematic review with 64 studies indicated that restrictions to reduce COVID-19 transmission affected PA and SB in the general population (7). In this scenario, studies indicate worsening mental health of MED students (14, 39). Since studies comparing different courses are still scarce, we compared a physically active course (PE) with a lower physically active course (MED). Our data align with other studies that evidenced that more time in MVPA and less time in SB may influence mental health (11, 41, 42).

It is well documented that the pandemic impacts sleep quality (43). When considering Sleep Quality (PSQI), a recent meta-analysis including 31 studies (*n* = 5,153) showed poor sleep quality in 60.4% of the sample analyzed (44), which is higher than our results in MED (54.0%) and PE (44.2%) students. Even though we verified significant differences between the components Habitual sleep efficiency, use of sleeping medication, and daytime dysfunction, the global PSQI score did not present differences. A recent study showed that the use of the internet, fear of leaving the house, and self-medication were the most common sleeping difficulties (45).

Finally, from a constructive perspective, our study allows us to speculate on the importance of organizing university environments, especially those used by medical students, to favor students reaching the PA recommendations and reducing their SB. In fact, a recent study highlighted the necessity of shared responsibility for students to perform more time in PA that resides at a political level, in welfare organizations, and in the educational institutions (46). One example of action is flexible learning spaces at school to allow students to spend less time sitting and more time standing and moving around (47), and the possibility of accessing outdoor and nature-based PA, as this exposure has a positive effect on different emotional parameters (e.g., anxiety, depression, stress) (48). Sporting activities can also be an important strategy to increase the PA time of students, as they provide health benefits for practitioners, and also lead to the attainment of the academic performance goals that educational institutions aspire to (49).

In relation to practical applications, considering the well-described relation of PA and anxiety and depression symptoms, which are common in medical students (frequent problems with anxiety and depression symptoms, not meeting the PA recommendation, and presenting high SB), we suggest there is a strong need to facilitate medical students to spend more time in PA and to reduce SB. We highlight the importance of providing strategies to increase the PA of university students, especially MED students, who showed poor physical activity levels (15). In this way, competitive university sports, which are already quite popular and offer recreational sports opportunities, represent alternatives to reduce SB and increase physical activity levels. Another possibility is encouraging the use of active behaviors, such as using stairs in university buildings, using spaces with dynamic characteristics for eating, classes, and computers (e.g., high benches, standing desks, or tables where the student participates in the theoretical actions while standing), and awareness of the importance of breaking prolonged sedentary behavior, among other actions. Finally, we suggest a strong need to facilitate medical students to spend more time on PA and to reduce SB.

### Strengths and Limitations

The strengths of this study included university students with different characteristics of PA (MED and PE), with high COVID-19 exposure in São Paulo City. Our study is not free of limitations. First, we included only one university. Second, despite the wide use of self-report tools for PA and SB measures, mental health may underestimate our findings. Finally, we could not investigate possible psychiatric conditions, such as a diagnosis of depression or anxiety disorders, in more detail.

## Conclusion

In conclusion, our results indicate that MED students spend less time in MVPA and more time in SB than PE students and presented worse mental health in the pandemic situation imposed by COVID-19.

## Data Availability Statement

The raw data supporting the conclusions of this article will be made available by the authors, without undue reservation.

## Ethics Statement

The studies involving human participants were reviewed and approved by Ethics and Research Committee of Santo Amaro University (approval number: 4.049.214). The patients/participants provided their written informed consent to participate in this study.

## Author Contributions

TM and KS: conceptualization, methodology, formal analysis, investigation, writing original draft, writing review and editing, and visualization. TG, AS, LN, and AB: writing original draft and writing review editing. SG: formal analysis and writing review editing. CF: conceptualization, methodology, writing original draft, and writing review editing. FR: writing review editing. LN: conceptualization, methodology, formal analysis, writing original draft, and writing review, supervision. All authors contributed to the article and approved the submitted version.

## Funding

The work was supported by the Grants 2020/08869-0, 2021/13699-9, 2021/14033-4 by São Paulo Research Foundation (FAPESP) in Brazil.

## Conflict of Interest

The authors declare that the research was conducted in the absence of any commercial or financial relationships that could be construed as a potential conflict of interest.

## Publisher's Note

All claims expressed in this article are solely those of the authors and do not necessarily represent those of their affiliated organizations, or those of the publisher, the editors and the reviewers. Any product that may be evaluated in this article, or claim that may be made by its manufacturer, is not guaranteed or endorsed by the publisher.
